# Knowledge gaps, psychological burden, and patient-reported symptom severity and daily functioning in autoimmune gastritis: a observational survey of Chinese patients

**DOI:** 10.3389/fmed.2026.1845745

**Published:** 2026-06-26

**Authors:** Miaoyan Chen, Shurong Hu

**Affiliations:** Department of Gastroenterology, The Second Affiliated Hospital, Zhejiang University School of Medicine, Hangzhou, China

**Keywords:** autoimmune gastritis, health education, patient perceived knowledge, patient-reported symptom severity and daily functioning, psychological impact

## Abstract

**Background:**

Autoimmune Gastritis (AIG) is a chronic inflammatory disorder characterized by autoimmune-mediated damage to the gastric mucosa, leading to gastric atrophy and may trigger serious complications such as vitamin B12 deficiency and pernicious anemia. This study aimed to evaluate knowledge gaps, psychological burden and patient-reported symptom severity and daily functioning (PR-SSDF) among Chinese patients with AIG.

**Methods:**

A questionnaire-based cross-sectional survey was distributed to 130 eligible AIG patients, of whom 113 participants fully completed the survey, achieving a response rate of 86.9%. The questionnaire collected information on sociodemographic characteristics, disease-related knowledge, lifestyle behaviors, as well as AIG-related impacts on patients’ daily activities and mental wellbeing.

**Results:**

The majority of respondents were female (66.36%), with the highest proportion in the 40–65 years age group (64.55%). A significant portion of patients had been diagnosed with AIG for less than a year (49.09%). Most resided in urban areas (63.64%). Perceived knowledge about AIG was found to be limited, with only a minority of patients achieved adequate disease comprehension. The majority of patients reported experiencing symptoms that affected their daily life and occupational activities. A substantial proportion of patients also reported experiencing extra-gastrointestinal symptoms and psychological distress following diagnosis. The majority of patients were covered by some form of medical insurance, with the largest group having employee medical insurance (87.27%).

**Conclusion:**

Our results reveal prominent self-perceived knowledge inadequacies, psychological distress, and impaired daily functioning among Chinese AIG patients, indicating an urgent need for tailored disease education and psychological support interventions.

## Introduction

Autoimmune Gastritis (AIG), also known as Type A Gastritis, is a progressive chronic inflammatory disorder that results in the deterioration of gastric parietal cells and the depletion of intrinsic factor, causing atrophy of the stomach (1). This condition leads to a reduction in gastric acid production, which can impair digestion and nutrient absorption, particularly of vitamin B12, potentially leading to pernicious anemia (2). Owing to frequent asymptomatic early-phase presentation, the overall prevalence of AIG remains poorly defined (3). Some studies suggest that it may be underdiagnosed due to insufficient awareness and reliance on endoscopy for diagnosis. A Chinese retrospective cohort reported an annual AIG detection rate of 0.9% based on combined histological and serological confirmation (4). Whereas a Japanese population-based screening study enrolling nearly 7,000 health check-up participants identified an AIG prevalence of 0.49% via endoscopic and serological methodologies (5). Epidemiologic data consistently demonstrate a prominent female predominance: adult women have a 2-3 fold higher AIG risk relative to men (6). The pathogenesis of AIG is multifactorial, involving genetic predisposition, environmental factors, dysregulated autoimmunity against targeted gastric antigens (7). The causal relationship between *Helicobacter pylori* (Hp) infection and AIG remains controversial. Individuals with HP infection exhibit an elevated risk of developing AIG, particularly in the context of certain genetic backgrounds. HP infection might potentially alter the immune response, thereby initiating or intensifying autoimmune reactions (8). Despite AIG being an immune mediated disease, there are currently no anti-inflammatory, immunosuppressive, or biologic therapies available for its treatment.

Individuals with AIG might suffer from upper gastrointestinal complaints including abdominal discomfort, abdominal bloating, and nausea, which can significantly affect their patient-reported symptom severity and daily functioning (PR-SSDF). In addition, the most frequent complication associated with AIG is vitamin B12 deficiency, which can result in megaloblastic anemia (1, 2). For patients with AIG, micronutrient supplementation is the primary therapeutic approach. While hematological changes are typically reversible with treatment, neurological effects might not be (1, 2). Addressing gastrointestinal symptoms presents a challenge due to the lack of specific treatments for these symptoms. Chronic conditions like AIG can lead to mental health concerns including anxiety and depression. The reported prevalence of impaired psychological status among AIG patients, including anxiety and depression, is 58% (9). This emphasizes the importance of exploring the multifaceted effects of AIG on patient well-being and developing targeted interventions to enhance PR-SSDF. The impact of AIG symptoms on PR-SSDF has not been adequately studied, even though these symptoms can significantly burden patients. Similar to patients with functional dyspepsia, those with AIG are more prone to experiencing disruptions in daily activities, and studies have shown that they have lower self-reported health, social functioning, and mental health (10). Long-term disease management also creates substantial financial burdens for both individual patients and healthcare systems. The present cross-sectional study was designed to comprehensively explore AIG’s physical, psychological and functional influences on Chinese patients, with three specific objectives: (1) to assess patients’ self-perceived disease knowledge AIG; (2) to quantify the disease-related psychological and daily functional impairments; (3) to identify factors influencing healthcare access and treatment adherence in this cohort.

## Methods

### Study design

**Figure 1:**
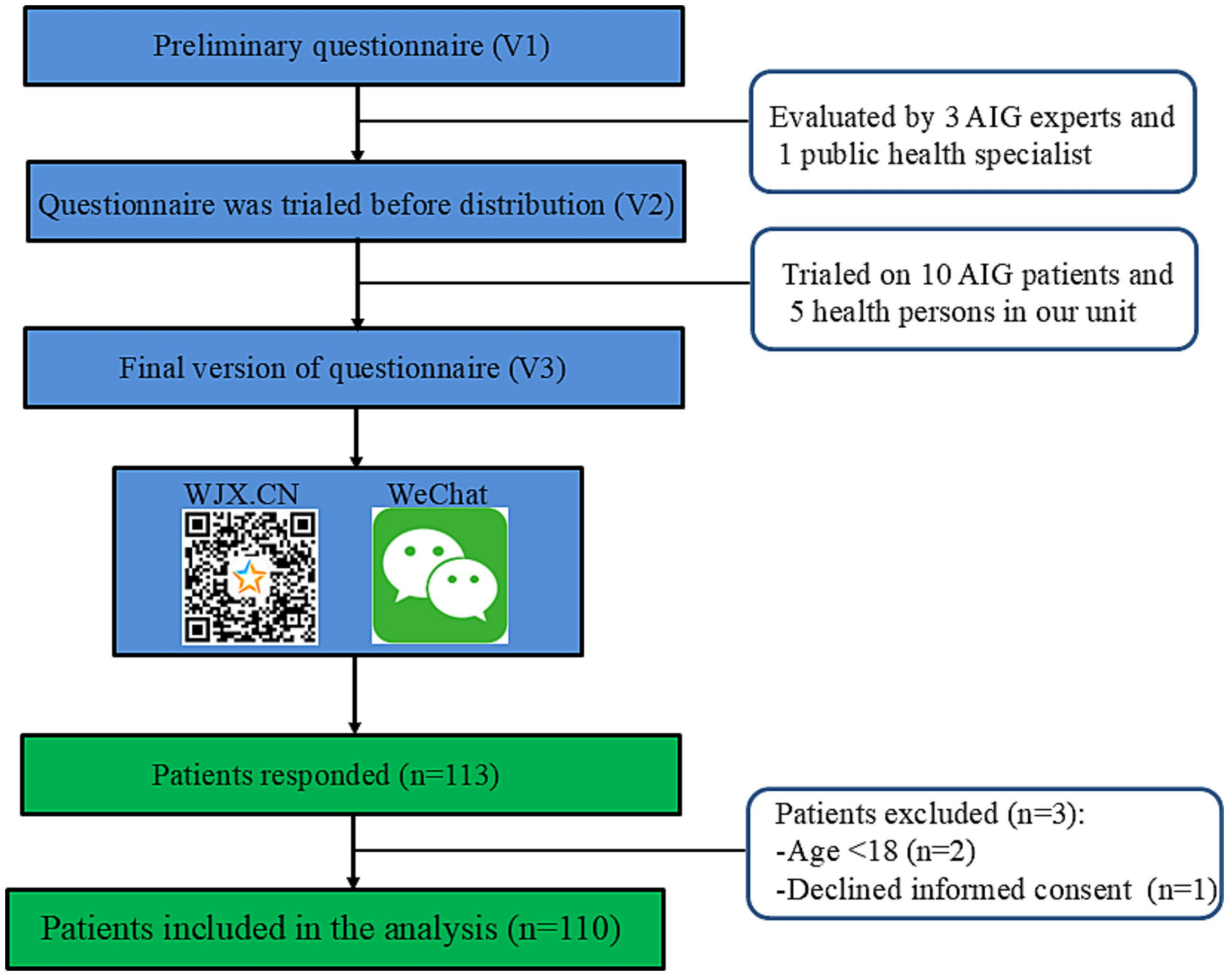
Flowchart of questionnaire development and patient recruitment process. The preliminary questionnaire (V1) was evaluated by three AIG experts and one public health specialist. The revised version (V2) was trialed on 10 AIG patients and 5 healthy individuals from our unit, leading to the final questionnaire (V3). The survey was distributed via WJX.CN and WeChat. A total of 113 patients responded; 3 were excluded (2 aged <18 years, 1 declined informed consent), resulting in 110 patients included in the analysis.

The research was carried out at the Second Affiliated Hospital of Zhejiang University School of Medicine from June 1, 2024, through August 31, 2024. All adult patients diagnosed with AIG at SAHZU were eligible for inclusion in the study. The criteria for inclusion comprised individuals aged 18 years or older who had been diagnosed with AIG through a combination of endoscopic observations, histological analysis of gastric biopsy samples, and serological screening for gastric autoantibodies (11, 12). Participants were asked to fill out an online survey. Patients were excluded if they could not comprehend survey questions, had treated psychiatric disorders, or presented with other coexisting autoimmune conditions. The questionnaire was developed in conjunction with healthcare professionals and experts in gastroenterology. It went through a pilot testing stage to guarantee the clarity, relevance, and comprehensiveness of the questions. Feedback from the pilot was utilized to improve the instrument. The questionnaire was made using Wenjuanxing, a free and accessible platform for survey design (13). Due to the web-based nature of the study, the traditional written consent form was replaced by an electronic consent process, which involved clicking “I agree” on the initial page of Wenjuanxing. In the electronic consent, participants were provided with a detailed information sheet that explained the purpose of the study, the procedures involved, potential risks and benefits, confidentiality assurances, and their right to withdraw at any time without penalty. Each participant was given ample time to review the information and ask questions before providing their consent. The questionnaire was distributed to a WeChat group dedicated to patient management, resulting in a response rate of 113 out of 130 patients who completed the online questionnaire. The flowchart was shown in Figure 1. This study was approved by the Institutional Review Board of the Ethics Committee at the Second Affiliated Hospital, School of Medicine, Zhejiang University, China (IR2024-0875). The approval also confirmed that our electronic consent process met the ethical standards and regulatory requirements for protecting participant rights.

### Patients’ characteristics

The survey was carefully designed to gage various aspects associated with AIG. The questionnaire was structured to address three key domains: perceived knowledge of AIG, psychological and PR-SSDF impacts, and healthcare access. It incorporated sections on basic demographic information, such as age, sex, educational level, and place of residence. To evaluate the participants’ understanding of AIG, the survey featured questions pertaining to their familiarity with the condition, its manifestations, treatment options, and prognosis. Additionally, the survey probed into the impact of AIG on the participants’ professional and personal spheres, including queries about workplace difficulties like frequent absences or diminished efficiency, as well as the effects on everyday activities such as social engagements and personal care routines. The survey also included questions aimed at assessing the emotional and psychological well-being of the participants, covering topics like stress, anxiety, depression, and strategies for coping.

### Statistical analysis

The statistical analyses were all performed with IBM SPSS software (version 25.0, Chicago, Illinois, United States). For continuous variables, the mean and standard deviation, or the inter-quartile range [IQR], were calculated to represent the central tendency and variability. Categorical variables were presented as frequencies or percentages, and the differences in these frequencies across groups were assessed using the Chi-square test or Fisher’s exact test, as appropriate. A two-sided *p*-value of below 0.05 was regarded as statistically significant for all analyses conducted.

## Results

Of the initial 130 distributed questionnaires (Supplementary file 1), 113 were returned, achieving a response rate of 86.9%, and 110 eligible patients were included in the final analysis (Figure 1). Baseline sociodemographic and clinical characteristics of enrolled subjects are presented below.

### Socio-demographic characteristics

Baseline socio-demographic characteristics were summarized in Table 1. The majority of respondents were female (66.36%), while 64.55% of patients fell within the 40–65 year age bracket. A substantial proportion of the participants resided in large cities with a population of over 1 million (63.64%). Regarding employment, full-time employees accounted for the largest proportion (64.55%). In terms of education, 54.55% held bachelor’s degrees and an additional 18.18% had postgraduate qualifications. Most respondents were married (90.91%), and 67.27% reported a monthly household income ranging from 5,000 to 20,000 Chinese Yuan (CNY).

**Table 1:** Sociodemographic and clinical characteristics of patients (*n* = 110).

Characteristic	Number of patients (*n*)	Percentage (%)
Sex, *n* (%)		
Male	37	33.64%
Female	73	66.36%
Age group (years)		
18–39	32	29.09%
40–65	71	64.55%
>65	7	6.36%
Marital status, *n* (%)		
Married	100	90.91%
Non-married	10	9.09%
Highest level of education completed, *n* (%)		
Primary or middle school certificate	14	12.72%
High school/secondary vocational school	16	14.55%
Bachelor’s level	60	54.55%
Master’s and doctorate’s level	20	18.18%
Self-reported monthly income, *n* (%)		
<2000 CNY	0	0%
2000–5000 CNY	12	10.91%
5,000–10,000 CNY	40	36.36%
10,000–20,000 CNY	34	30.91%
>20,000 CNY	24	21.82%
Working condition, *n* (%)		
Full-time	71	64.55%
Part-time	4	3.64%
Student/unemployment/retirement/others	35	31.81%
Duration of disease, *n* (%)		
Less than 1 year	54	49.09%
1–3 years	47	42.73%
3–10 year	8	7.27%
More than 10 years	1	0.91%
Usual place of residence		
Large city (with a population of over one million)	70	63.64%
Medium-sized city (with a population of 200,000 to 1 million)	26	23.63%
Township	14	12.73%
Health insurance coverage		
Self-pay	1	0.91%
Employee medical insurance	96	87.27%
Resident medical insurance	12	10.91%
Rural cooperative medical scheme	4	3.64%
Commercial insurance	11	10%

### Health behaviors and lifestyle

Most patients (70.91%) maintained a balanced light diet combining vegetables and meat; 13.64% preferred spicy food, and 18.18% regularly consumed tea, coffee or carbonated beverages. In terms of lifestyle, 48.18% of the participants reported having regular sleep patterns, while 20% admitted to irregular sleep habits and frequent staying up late. In addition, 3.64% of AIG patients have a history of smoking, and 6.36% of AIG patients have a history of drinking.

### Perceived knowledge and disease perception of AIG

**Figure 2:**
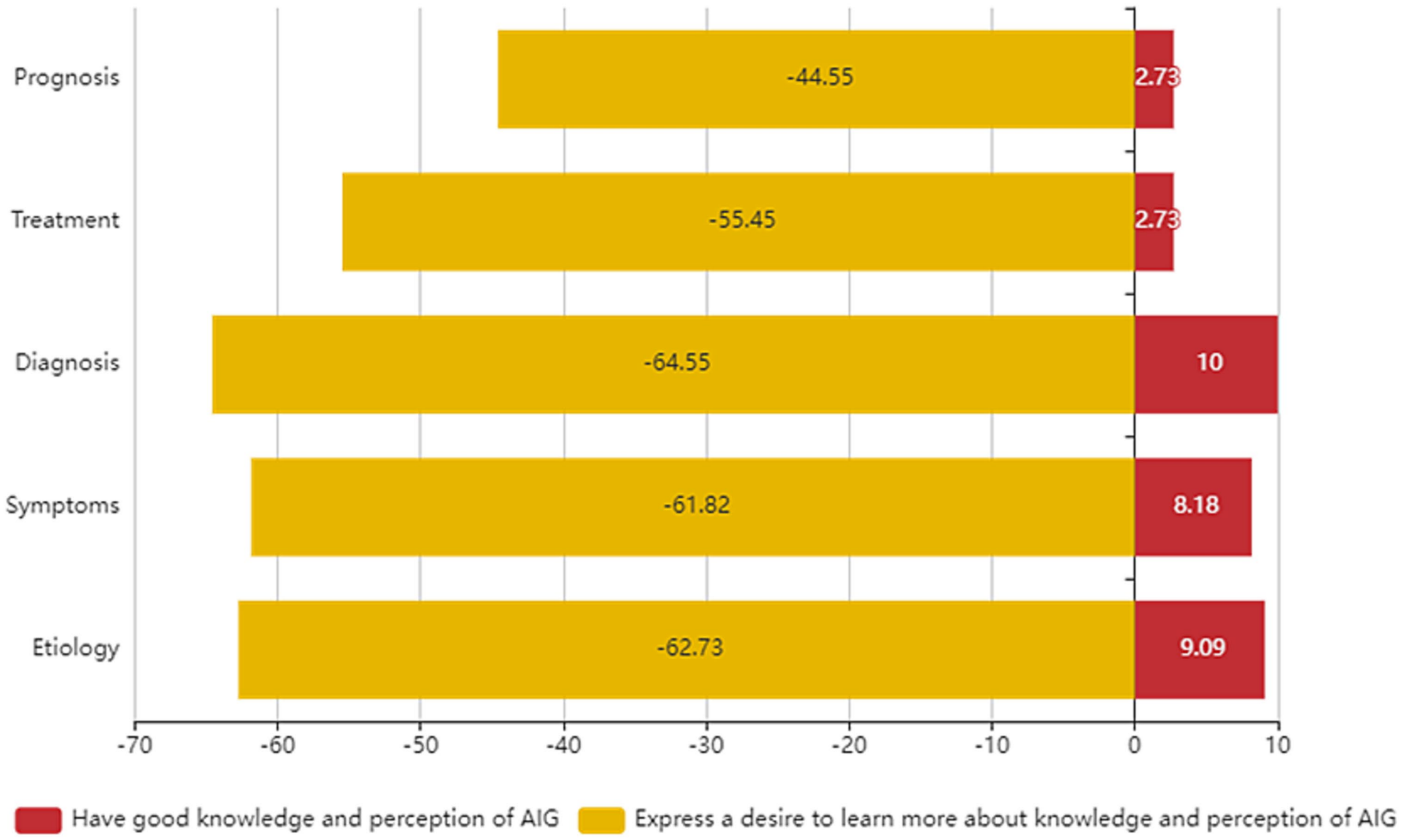
Patients’ perceived knowledge and desire for information about AIG. For each domain (etiology, symptoms, diagnosis, treatment, prognosis), the bar chart shows the percentage of patients who reported having good perceived knowledge and those who expressed a desire to learn more. Numerical values are indicated on the bars.

Self-perceived understanding of AIG remained poor across all domains. Less than 10% of respondents reported adequate awareness regarding AIG etiology and pathogenesis (9.09%), clinical manifestations (8.18%), diagnostic methods (10%), available therapies (2.73%), and long-term prognosis (2.73%) (Figure 2). Notably, over half of participants were eager to acquire supplementary disease-related knowledge: 62.73% for etiology and pathogenesis, 61.82% for disease symptoms, 64.55% for diagnostic methods, 55.45% for treatment strategies, and 44.55% for prognostic information.

### Impact of AIG on daily life and occupational function

A substantial number of participants reported experiencing postprandial bloating and other digestive symptoms that affected their daily life and work (65.86%). Additionally, 38.18% reported experiencing extra-gastrointestinal symptoms such as dizziness, numbness in limbs, mood instability, and menstrual irregularities, which also impacted their PR-SSDF.

### Healthcare access and medical insurance

Regarding healthcare access, as shown in Table 1, 87.27% of the respondents were covered by employee basic medical insurance, 10.91% by urban resident medical insurance, and 3.64% by rural cooperative medical insurance. An extra 10.00% purchased supplementary commercial medical insurance, and only 0.91% reported having no insurance coverage (Table 1). Patients mainly obtained AIG-related health information via three online channels: professional medical websites (30%), WeChat official accounts (42.73%), and Baidu search engine (44.55%). When evaluating online information quality, 38.18% rated online resources as very helpful and another 42.73% as moderately helpful.

### Psychological impacts following AIG diagnosis

**Figure 3:**
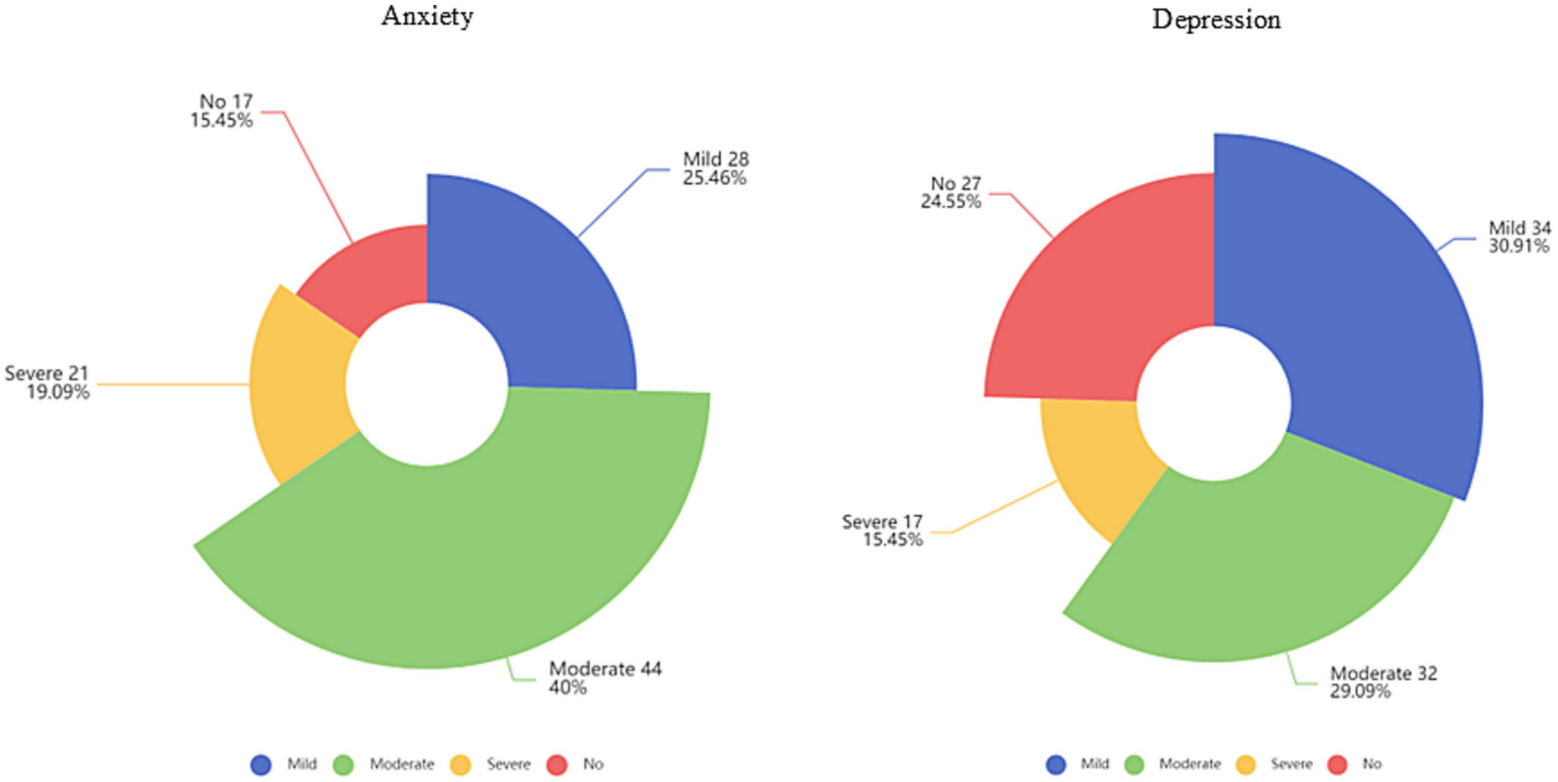
Levels of anxiety and depression among AIG patients. The grouped bar chart displays the proportion of patients reporting no, mild, moderate, or severe symptoms of anxiety and depression during the initial period after diagnosis. Percentages are shown on the bars.

Shortly after AIG confirmation, a significant number of participants developed anxiety, irritability, and excessive worrying, with 59.09% indicating moderate to severe levels of these symptoms (Figure 3). In terms of depressive symptoms, 30.91% presented mild depression and 15.45% severe depressive complaints within the first week post-diagnosis (Figure 3).

### Treatment and follow-up adherence

**Figure 4:**
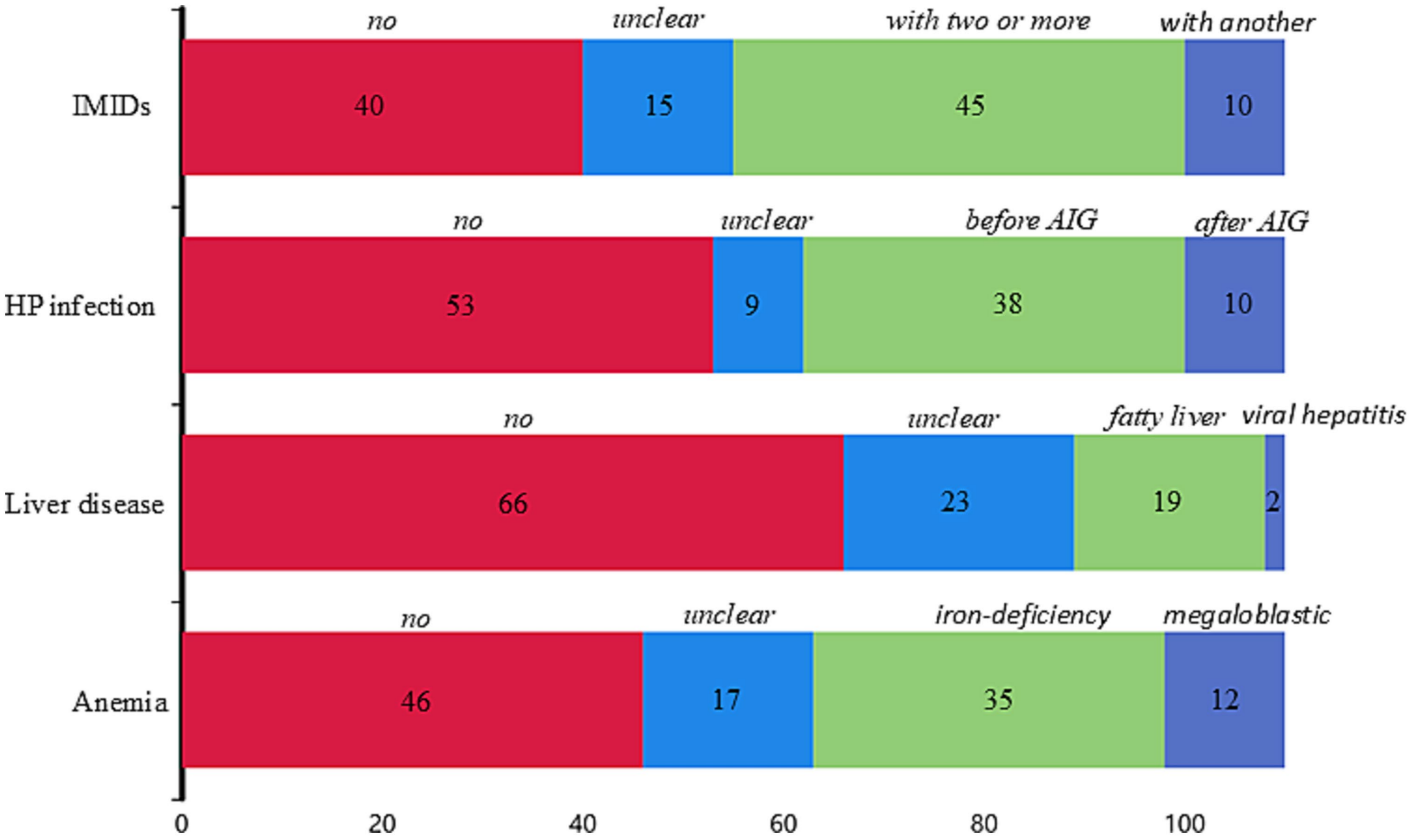
Comorbidities in AIG patients. The bar chart shows the number of patients with anemia, liver disease, *H. pylori* infection, and other immune-mediated inflammatory diseases (IMIDs). Exact counts are displayed on the bars.

Regarding hematologic complications, 31.82% (*n* = 35) were diagnosed with iron-deficiency anemia, and 10.91% (*n* = 12) with megaloblastic anemia (Figure 4). Concomitant liver disorders consisted of non-alcoholic fatty liver disease (17.27%, *n* = 19) and viral hepatitis (1.82%, *n* = 2). For *Helicobacter pylori* infection, 34.55% (*n* = 38) received an Hp diagnosis prior to AIG onset and 9.09% (*n* = 10) were diagnosed after AIG identification (Figure 4). In addition, 45 patients were complicated with other Immune-Mediated Inflammatory Diseases (IMIDs) including autoimmune hepatitis, inflammatory bowel disease, among whom 10 individuals suffered from two or more distinct IMIDs (Figure 4). Most participants (84.55%) strictly complied with physician-recommended follow-up protocols including regular blood tests and endoscopic surveillance, while only 1.82% declined scheduled follow-up arrangements.

## Discussion

The survey results provide real-world sociodemographic features, lifestyle characteristics and self-perceived disease cognition among Chinese AIG patients prior to standardized health education. Consistent with existing epidemiologic evidence, female predominance was confirmed within our cohort, which matches the well-established higher AIG susceptibility in women (6). The age distribution of respondents indicates that AIG is more common in middle-aged and older adults, aligning with the known association between age and the development of autoimmune diseases. The high proportion of respondents residing in large cities is likely attributable to superior local medical resources facilitating timely AIG screening and definitive diagnosis (3, 14). Our survey demonstrated prominent deficits in patients’ self-perceived AIG knowledge: fewer than 10% of respondents claimed to have a comprehensive understanding of the disease’s etiology, symptoms, diagnostic methods, treatment options, and prognosis. Insufficient disease awareness potentially delays early intervention, hinders self-management capacity and lowers long-term treatment adherence (5, 15). Meanwhile, the overwhelming demand for additional disease education highlights an urgent need for targeted, individualized health promotion programs to optimize patients’ disease cognition and self-care behavior.

The high prevalence of digestive symptoms such as postprandial bloating and the reported impact on daily life underscore the significant burden of AIG on patients’ PR-SSDF (9). The reported extra-gastrointestinal symptoms, including dizziness and mood instability, further emphasize the systemic nature of AIG and its broader impact on patient well-being. These findings suggest that AIG is not merely a localized gastric condition but has broader implications for overall health and well-being. The impact on daily life reinforces the importance of holistic care that addresses not only the physical symptoms but also the psychosocial aspects of living with AIG. In addition, the majority of respondents also had some form of medical insurance, which is crucial for accessing diagnostic and treatment services for AIG. However, the small percentage of uninsured individuals may face financial barriers to care, suggesting a need for advocacy and support to ensure equitable access to healthcare.

Patients’ reliance on various online platforms for health information is evident, with a significant number turning to WeChat public accounts, medical professional websites, and search engines like Baidu (16, 17). The mixed perceptions of the helpfulness of online health information highlight the need for accurate, accessible, and patient-friendly educational resources. It also emphasizes the role of healthcare providers in guiding patients to reliable sources of information. In addition, the prevalence of anemia and liver disease among respondents is noteworthy, as these conditions can complicate AIG management and contribute to increased morbidity (18). The high rate of *Helicobacter pylori* infection in this population also suggests that patients with AIG may be at greater risk for other gastrointestinal infections, warranting further investigation and appropriate preventive measures (19, 20).

The psychological impact of AIG diagnosis was significant, with many respondents experiencing anxiety, irritability, and depressive symptoms (1, 2). These findings underscore the importance of integrating mental health support into the care plan for AIG patients. Early psychological intervention may improve patient adaptation and long-term outcomes. The high rate of agreement and compliance with the follow-up plans, including blood tests and gastroscopy, indicates a generally positive attitude toward medical management (1, 2). However, the small percentage of non-compliant patients may represent a group at risk for poor disease control, warranting further investigation into the barriers to adherence.

Our study had some shortcomings. First, the study was a single-center cross-sectional survey; cross-sectional design precludes causal inference, and self-reported questionnaires inherently carry potential recall bias. Second, convenience sampling may introduce selection bias. Third, a limitation of our study was the absence of validated psychometric instruments and scoring systems, as well as the lack of an objective test to assess patients’ objective knowledge. Finally, due to the survey’s focus on patient-reported outcomes (e.g., objective knowledge, lifestyle, psychological impact), we did not systematically collect granular clinical data such as specific endoscopic findings, histopathological scores or individualized treatment regimens. Future studies will incorporate these clinical parameters to better correlate patient experiences with objective disease severity.

## Conclusion

In conclusion, Chinese AIG patients exhibit widespread insufficient self-perceived disease knowledge alongside strong willingness to receive systematic health education. AIG-related physical symptoms substantially disrupt patients’ daily living and occupational activities, highlighting the critical need for timely psychological support after confirmed diagnosis Furthermore, the majority of participants have access to medical insurance and are compliant with the recommended treatment and follow-up schedules.

## Data Availability

The original contributions presented in the study are included in the article/Supplementary material, further inquiries can be directed to the corresponding author.
