# Supplementary material for: Knowledge gaps, psychological burden, and patient-reported symptom severity and daily functioning in autoimmune gastritis: a observational survey of Chinese patients
Source: Front Med (Lausanne). 2026 Jun 26;13:1845745. doi: 10.3389/fmed.2026.1845745 (PMC13349835; doi:10.3389/fmed.2026.1845745)
Supplement: Supplementary file 1 [file Supplementary_file_1.docx]

**Survey on the Situation of Patients with Autoimmune Gastritis (AIG)**

I confirm that I have read and understood the informed consent form for this study, voluntarily agree to be a subject, and consent to the use of my data for the publication of this study.[Single choice]

- Agree
- Disagree (end the survey)

**I. General Information**

1. Gender: [Single choice]
   a) Male
   b) Female
2. Age (years): [Single choice]
   a) <18
   b) 18–39
   c) 40–65
   d) >65
3. How long have you been diagnosed with AIG? [Single choice]
   a) <1 year
   b) 1–3 years (excluding 3 years)
   c) 3–10 years (excluding 10 years)
   d) ≥10 years
4. Where do you usually live? [Single choice]
   a) Large city (population >1 million)
   b) Medium/small city (population 200,000–1 million)
   c) Town or village
5. Your employment status: [Single choice]
   a) Full-time employed
   b) Part-time employed
   c) Student
   d) Unemployed
   e) Retired
   f) Other
6. Your current occupation: [Single choice]
   a) Head of government, party, enterprise, or public institution
   b) Professional and technical staff
   c) Clerical and related personnel
   d) Commercial and service personnel
   e) Agricultural, forestry, animal husbandry, fishery, and water conservancy production personnel
   f) Production and transport equipment operators and related personnel
   g) Military personnel
   h) Other
7. Education level: [Single choice]
   a) Illiterate
   b) Primary school
   c) Junior high school
   d) Senior high school or secondary school
   e) University
   f) Postgraduate or above
8. Marital status: [Single choice]
   a) Married
   b) Unmarried
   c) Divorced
   d) Widowed
   e) Other
9. Your current dietary and lifestyle habits: [Multiple choice]
   a) Light diet, mainly vegetarian
   b) Light diet, mainly meat/seafood
   c) Light diet, balanced meat and vegetables
   d) Prefer heavy flavors (e.g., spicy hot pot, barbecue)
   e) Prefer raw/cold foods
   f) Enjoy tea/coffee/beverages
   g) Enjoy smoked or pickled foods
   h) Regular routine, no staying up late
   i) Irregular routine, often stay up late
   j) Regular eating habits
   k) Irregular eating habits
   l) Smoking
   m) Drinking alcohol
   n) No smoking or drinking history
   o) Other
10. Monthly household income (approx.): [Single choice]
    a) <2,000 RMB
    b) 2,000–5,000 RMB (excluding 5,000)
    c) 5,000–10,000 RMB (excluding 10,000)
    d) 10,000–20,000 RMB (excluding 20,000)
    e) ≥20,000 RMB
11. Do you have medical insurance? (Multiple choices) [Multiple choice]
    a) Employee medical insurance
    b) Resident medical insurance
    c) Rural cooperative medical insurance
    d) Commercial insurance
    e) None

**II. Your Understanding of AIG**

1. Do you understand the etiology and pathogenesis of AIG? [Single choice]
   a) Understand
   b) Somewhat understand, want to know more
   c) Somewhat understand, do not want to know more
   d) Do not understand, want to know
   e) Do not understand, and do not want to know
2. Do you understand the impact of diet and lifestyle on AIG? [Single choice]
   a) Understand
   b) Somewhat understand, want to know more
   c) Somewhat understand, do not want to know more
   d) Do not understand, want to know
   e) Do not understand, and do not want to know
3. Do you understand the symptoms of AIG? [Single choice]
   a) Understand
   b) Somewhat understand, want to know more
   c) Somewhat understand, do not want to know more
   d) Do not understand, want to know
   e) Do not understand, and do not want to know
4. Do you know the diagnostic methods for AIG? [Single choice]
   a) Understand
   b) Somewhat understand, want to know more
   c) Somewhat understand, do not want to know more
   d) Do not understand, want to know
   e) Do not understand, and do not want to know
5. Do you understand the treatment methods for AIG? [Single choice]
   a) Understand
   b) Somewhat understand, want to know more
   c) Somewhat understand, do not want to know more
   d) Do not understand, want to know
   e) Do not understand, and do not want to know
6. Do you know the prognosis of AIG? [Single choice]
   a) Understand
   b) Somewhat understand, want to know more
   c) Somewhat understand, do not want to know more
   d) Do not understand, want to know
   e) Do not understand, and do not want to know
7. Do you understand the complications of AIG? [Single choice]
   a) Understand
   b) Somewhat understand, want to know more
   c) Somewhat understand, do not want to know more
   d) Do not understand, want to know
   e) Do not understand, and do not want to know
8. Do you know that AIG can co-exist with other immune-related diseases? [Single choice]
   a) Understand
   b) Somewhat understand, want to know more
   c) Somewhat understand, do not want to know more
   d) Do not understand, want to know
   e) Do not understand, and do not want to know
9. Do you hope and are you willing to receive immunosuppressive therapy (etiologic treatment) for AIG? [Single choice]
   a) Hope and willing to try
   b) Hope, but not willing to try without evidence-based medical evidence
   c) Hope, but not willing to try until recommended by guidelines
   d) Do not hope, not willing
10. Do you have online sources for AIG information? Which do you mainly follow? (Multiple choice) [Multiple choice]
    a) Professional medical websites
    b) WeChat public accounts
    c) Mini-programs
    d) Weibo
    e) Douyin (TikTok)
    f) Bilibili
    g) Xiaohongshu (Little Red Book)
    h) Patient support groups
    i) Baidu
    j) Other
    k) None
11. Do you find online information helpful? [Single choice]
    a) Very helpful
    b) Helpful, but not very
    c) Slightly helpful
    d) Not helpful
    e) Not helpful, and it increases worry or causes trouble
12. Do you have a familiar medical team for AIG? [Single choice]
    a) Yes, and do not want to look for another
    b) Yes, but want to learn about other teams
    c) Yes, but want to change teams
    d) No, want to find one
    e) No, and do not want to find one

**III. Impact of AIG on Work, Life, and Psychology**

1. Do you have postprandial bloating or other dyspeptic symptoms? Do they affect your work/life? [Single choice]
   a) Yes, severely affect
   b) Yes, sometimes affect
   c) Yes, but do not affect
   d) No, no impact
2. Do you have extra-gastrointestinal symptoms such as dizziness, limb numbness, emotional instability, or menstrual irregularities (for women)? Do they affect your work/life? [Single choice]
   a) Yes, 2 or more symptoms, severely affect
   b) Yes, 2 or more symptoms, sometimes affect
   c) Yes, single symptom, sometimes affect
   d) Yes, but no impact
   e) No, no impact
3. Has a doctor diagnosed you with anemia? [Single choice]
   a) Yes, iron deficiency anemia
   b) Yes, megaloblastic anemia
   c) Yes, but type unknown
   d) Not sure
   e) No
4. Has a doctor diagnosed you with liver disease? [Single choice]
   a) Yes, fatty liver with normal liver function
   b) Yes, fatty liver with abnormal liver function
   c) Yes, other liver disease
   d) Not sure
   e) No
5. Has a doctor diagnosed you with H. pylori infection? (Multiple choices) [Multiple choice]
   a) Yes, before AIG diagnosis
   b) Yes, after AIG diagnosis
   c) Yes, but difficult to eradicate
   d) Not sure
   e) No
6. Do you have other immune-related diseases (e.g., thyroiditis, type 1 diabetes, vitiligo, alopecia, rheumatoid arthritis)? [Single choice]
   a) Yes, 2 or more
   b) Yes, 1 type
   c) Not sure, haven't paid attention
   d) No
7. During the first 2 weeks after diagnosis, how often were you bothered by problems such as feeling nervous, anxious, or irritable; uncontrollable worry; excessive worry about things; inability to relax; restlessness; getting annoyed easily; fear that something terrible might happen? [Single choice]
   a) None
   b) Mild (<3 days)
   c) Moderate (3–7 days)
   d) Severe (>7 days)
   e) Pre-existing anxiety/depression worsened
   f) Pre-existing anxiety/depression unaffected
8. During the first week after diagnosis, how often were you bothered by problems such as lack of interest or pleasure in doing things; feeling down, depressed, or hopeless; trouble falling asleep, staying asleep, or sleeping too much; feeling tired or having little energy; poor appetite or overeating; feeling bad about yourself, feeling like a failure or that you let your family down; trouble concentrating (even on TV), memory decline; moving or speaking slowly that others notice; thoughts of death or hurting yourself? [Single choice]
   a) None (<1 day)
   b) Mild (1–3 days)
   c) Moderate (4 days)
   d) Severe (5–7 days)
   e) Pre-existing anxiety/depression worsened
   f) Pre-existing anxiety/depression unaffected
   g) Other
9. In the past 2 weeks, how often were you bothered by problems such as feeling nervous, anxious, or irritable; uncontrollable worry; excessive worry about things; inability to relax; restlessness; getting annoyed easily; fear that something terrible might happen? [Single choice]
   a) None
   b) Mild (<3 days)
   c) Moderate (3–7 days)
   d) Severe (>7 days)
   e) Pre-existing anxiety/depression worsened
   f) Pre-existing anxiety/depression unaffected
10. In the past week, how often were you bothered by problems such as lack of interest or pleasure in doing things; feeling down, depressed, or hopeless; trouble falling asleep, staying asleep, or sleeping too much; feeling tired or having little energy; poor appetite or overeating; feeling bad about yourself, feeling like a failure or that you let your family down; trouble concentrating (even on TV), memory decline; moving or speaking slowly that others notice; thoughts of death or hurting yourself? [Single choice]
    a) None (<1 day)
    b) Mild (1–3 days)
    c) Moderate (4 days)
    d) Severe (5–7 days)
    e) Pre-existing anxiety/depression worsened
    f) Pre-existing anxiety/depression unaffected
    g) Other
11. What do you think is the reason for changes in your emotional state? (Multiple choices) [Multiple choice]
    a) I have never had any emotional fluctuations
    b) No symptoms at all
    c) Doctor’s diagnosis and treatment
    d) Stable test results
    e) Still no good treatment options
    f) Disease progression or complications (e.g., tumor found)
    g) Other
12. Do you agree with and follow the doctor’s follow-up plan, including blood tests and gastroscopy? [Single choice]
    a) Agree and follow
    b) Agree but do not follow
    c) Have my own thoughts, and follow after shared decision-making with the doctor
    d) Disagree, do not follow, and do things my own way
